# Economic evaluation of lifestyle interventions in infertility management: A systematic review

**DOI:** 10.1371/journal.pone.0306419

**Published:** 2024-08-23

**Authors:** Suvijak Untaaveesup, Brandon Chongthanadon, Chayanis Kositamongkol, Pochamana Phisalprapa, Krasean Panyakhamlerd, Vitaya Titapant

**Affiliations:** 1 Phaholpolpayuhasena Hospital, Kanchanaburi, Thailand; 2 Faculty of Medicine Siriraj Hospital, Mahidol University, Bangkok, Thailand; 3 Faculty of Medicine Siriraj Hospital, Department of Medicine, Division of Ambulatory Medicine, Mahidol University, Bangkok, Thailand; 4 Faculty of Medicine, Department of Obstetrics and Gynecology, Chulalongkorn University, Bangkok, Thailand; 5 Faculty of Medicine, Department of Obstetrics and Gynecology, Siriraj Hospital, Mahidol University, Bangkok, Thailand; University Hospital of Münster, GERMANY

## Abstract

**Introduction:**

Infertility, a global concern affecting both sexes, is influenced by modifiable and non-modifiable risk factors. While the literature predominantly underscores the clinical- and cost-effectiveness of lifestyle interventions in the realm of infertility treatment, a holistic compilation analysing the economic dimensions of such interventions is lacking. This systematic review aimed to fill this gap by evaluating the economic facets of lifestyle interventions in the management of infertility.

**Methods:**

An exhaustive search was conducted within the PubMed, Embase, and Scopus databases from their inception to February 2024. The aim was to find articles related to the economic aspects of lifestyle interventions in infertility management. These included clinical studies covering economic outcomes and economic evaluations. The Drummond Checklist was used to assess the quality of the included studies.

**Results:**

From an initial yield of 7555 articles, five studies were deemed eligible for inclusion, comprising three cost-effectiveness analyses, one prospective cohort study and a randomized controlled trial, all of which were undertaken in high-income countries (the Netherlands, Australia and Japan). These studies included patients receiving infertility treatments for conditions such as unexplained infertility, polycystic ovary syndrome, ovulation disorders, or mild male infertility, inclusive of individuals with and without obesity. The women who participated in these studies were up to 45 years of age. The findings suggested that integrating lifestyle intervention programmes tends to enhance pregnancy and live birth outcomes. These programmes encompass coaching, psychological or behavioural guidance, nutritional adjustments, exercise regimes, weight management, smoking cessation and mindfulness techniques. Moreover, these interventions are likely to be more cost-effective than standard infertility care.

**Conclusion:**

For couples embarking on infertility treatments, the integration of lifestyle interventions into their management strategy not only fosters clinical benefits but also represents a cost-effective alternative to conventional care, particularly within high-income settings.

## Introduction

Infertility, defined as the inability to conceive following 12 months of regular, unprotected sexual intercourse, constitutes a significant global health issue impacting both sexes [1,2]. Infertility encompasses a range of risk factors, both modifiable, such as lifestyle, and non-modifiable, such as age and genetics [2,3]. Distinctions are made between primary infertility, where couples have not had children, and secondary infertility, where couples have previously conceived. The incidence of primary infertility decreases with age, while that of secondary infertility increases. Specifically, the prevalence of primary infertility in individuals aged 20–24 years is 2.7%, compared with 1.6%–1.7% in those aged 30–44 years. In stark contrast, the incidence of secondary infertility increases from 2.6% in younger individuals to 27.1% in older individuals [4]. According to an extensive analysis conducted by the World Health Organization between 1990 and 2021, the estimated lifetime prevalence of infertility was reported to be 17.5%, with a period prevalence of 12.6% [5].

Various treatments for infertility are available, including medications, assisted reproductive technologies and lifestyle interventions, which serve as effective primary measures. Modifiable lifestyle factors such as diet, smoking, substance abuse, caffeine intake and obesity detrimentally impact fertility [2,6]. A systematic review highlighted that infertility outcomes are contingent upon the modification of risk factors preceding pregnancy [7]. A study involving infertile patients with obesity who were subjected to assisted reproductive technologies divided participants into five groups. Four groups received lifestyle intervention programmes focused on weight reduction, pre-pregnancy lifestyle modifications and nutritional improvements, whereas the fifth group was not subjected to any interventions. The findings indicated an enhanced pregnancy rate in the groups that received intervention through phone and online communication [8]. Moreover, in a randomized controlled trial encompassing 793 patients undergoing *in vitro* fertilization treatment, 369 individuals in the intervention group who benefited from a coaching programme addressing nutritional and behavioural changes demonstrated a superior pregnancy rate in comparison with those receiving standard *in vitro* fertilization treatment [9]. Additionally, recommendations suggest that fertility rates can be improved through smoking cessation or reduction, weight loss and a diet rich in vegetables and low in red meat [6].

A recent study from France revealed that the economic burden associated with medication for infertility treatment among women aged 18 to 50 years was estimated at approximately 70 million euros per 10 000 women [10]. The analysis of expenditure breakdown indicated that medical costs constituted 6%–47% of the total cost, while technical acts and biology accounted for 5%–19% and 5%–32% of the total cost, respectively. Earlier studies have proposed that the incorporation of lifestyle intervention programmes alongside conventional infertility treatments could result in both cost savings and enhanced cost-effectiveness [8,9,11,12].

Nevertheless, the present body of evidence demonstrates inconsistencies and a lack of comprehensiveness concerning the economic evaluation of lifestyle intervention treatments. Hence, this systematic review aimed to offer a comprehensive assessment and evaluation of the economic dimension of lifestyle interventions in infertility treatment.

## Materials and methods

This systematic review was conducted in accordance with the Preferred Reporting Items for Systematic Reviews and Meta-Analyses (PRISMA) guidelines [13], as detailed in **S1 File**. The review was registered with the International Platform of Registered Systematic Review and Meta-Analysis Protocols (protocol number: INPLASY202410010) [14].

### Search strategy

Two researchers (SU and BC) independently executed a thorough search across three databases—PubMed, Embase and Scopus—from their inception until February 2024. This search aimed to identify relevant articles pertaining to the economic evaluation of lifestyle interventions in infertility treatment. The search strategy incorporated terms related to economic evaluations, including cost, cost-minimization, cost-effectiveness, cost-benefit and cost-utility analyses, alongside other terms related to infertility and subfertility, attitudes towards health, behavioural therapy and patient education. Details of the specific terms used and the search strategy are presented in **S2 File**. Additionally, the reference lists of the included studies were meticulously reviewed to ensure that the present systematic review was comprehensive.

### Inclusion and exclusion criteria

The eligibility criteria mandated the incorporation of economic evaluations, such as cost-minimization, cost-effectiveness, cost-benefit or cost-utility analyses, as well as clinical studies reporting economic outcomes, irrespective of population size. Studies were included if they documented economic outcomes alongside the efficacy, effectiveness or safety of lifestyle interventions for managing infertility, in contrast to patients who either received no intervention or were subjected to non-lifestyle intervention programmes. Lifestyle interventions were characterized as non-medical approaches, including weight reduction, educational programmes, and diets low in fat or energy. All studies had to be published in the English language. Exclusion criteria encompassed case reports, case series, systematic reviews, meta-analyses, review articles, non-English language publications, and studies failing to report on economic evaluation outcomes.

Two authors (SU and BC) independently conducted an initial screening of titles and abstracts of relevant literature using Covidence, an online tool designed for article screening in systematic reviews. Subsequently, a second round of review and a comprehensive full-text literature assessment were carried out via Covidence. Any discrepancies encountered were deliberated with additional investigators (CK and PP) until a consensus was achieved.

### Outcomes of interest

The focal outcomes were pregnancy-related metrics (namely, pregnancy and live birth rates) and economic indicators (total cost, cost per pregnancy, cost per delivery, cost per live birth and the incremental cost-effectiveness ratio). These were delineated in accordance with the definitions provided within each study included in the review.

### Data extraction and quality assessment

For data extraction, the designated form included the following details from eligible studies: first author, publication year, country of origin, study design (specifying economic or clinical focus), participant demographics, intervention specifics, participant numbers and mean age. The clinical outcomes of interest covered pregnancy-related metrics, specifically pregnancy and live birth rates. The economic outcomes captured were total cost, cost per pregnancy, cost per live birth and the incremental cost-effectiveness ratio. Authors SU and BC independently performed the data extraction, which was then verified by two additional researchers (CK and PP) to ensure accuracy and completeness.

To evaluate the quality of the included studies, two researchers independently used the Drummond Checklist, a 10-point tool for assessing the quality of economic evaluation studies. The quality interpretations were categorized as follows: scores below 6 indicated poor quality, scores between 6 and 8 suggested moderate quality, and scores above 8 denoted high quality [15]. The Drummond Checklist is detailed in **S3 File**.

## Results

**Fig 1:**
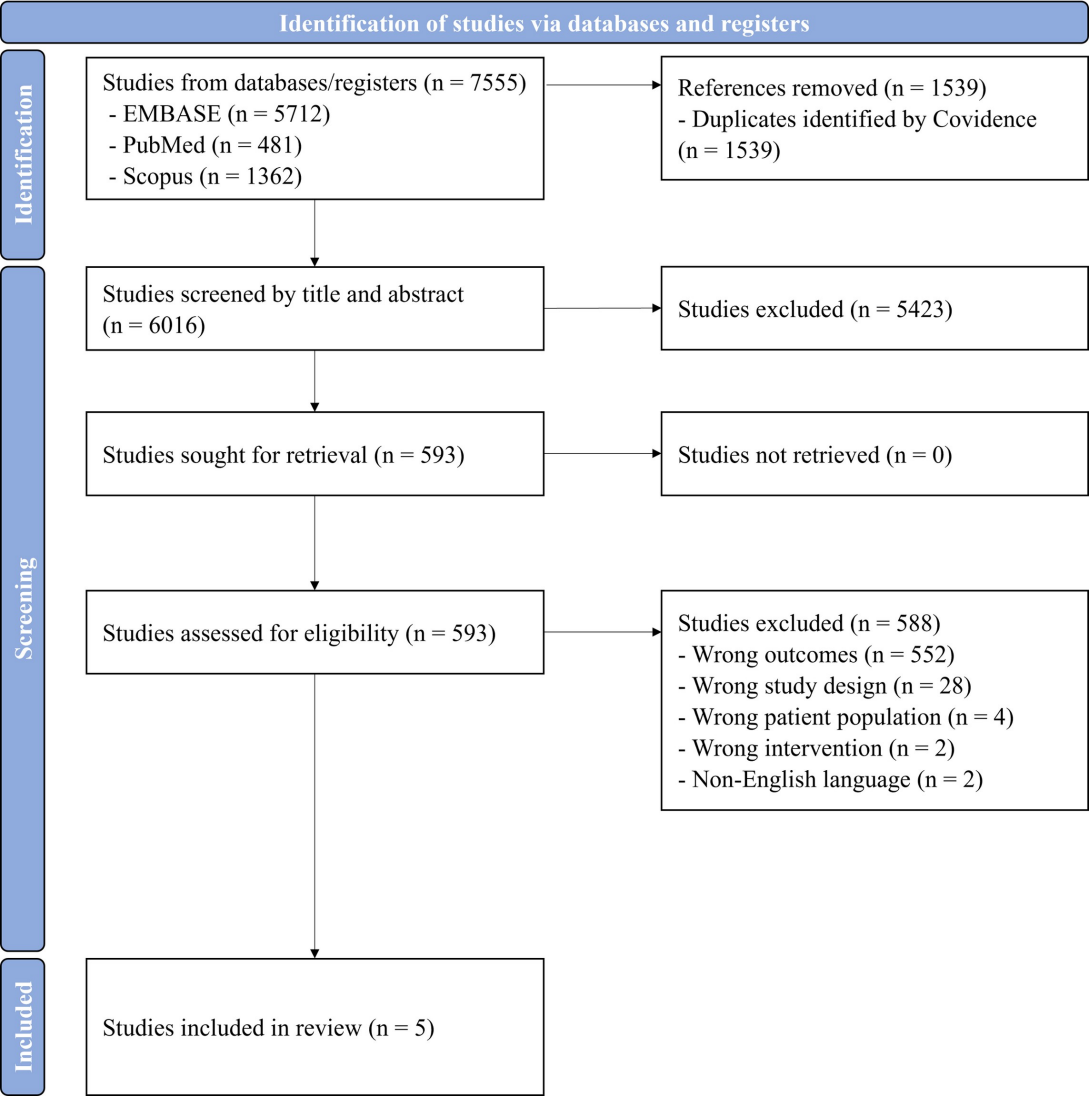
Flow diagram of article search and screening methodology.

The search yielded 7555 articles: 5712 from Embase, 481 from PubMed, 1362 from Scopus and none from the manual searches of reference lists. Through the use of Covidence, 1539 duplicate articles were identified and removed. The first screening round led to the exclusion of 5423 articles for not meeting the eligibility criteria. Subsequently, 593 full-text articles underwent a second-round review, with 588 articles excluded for various reasons: inappropriate outcomes (n = 552), inapplicable study design (n = 28), unsuitable study population (n = 4), incorrect intervention (n = 2) and non-English language (n = 2). Ultimately, five articles focusing on the economic evaluation of lifestyle interventions in infertility management were included [8,9,11,12,16]. These selected studies had moderate-to-high quality according to the Drummond Checklist, as detailed in **S3 File**. The sequential process of the article search and selection is illustrated in the **(Fig 1)**.

### Study characteristics

The review encompassed five studies that reported economic outcomes. They comprised three cost-effectiveness analyses [8,9,16], one randomized controlled trial [11] and one prospective cohort study [12]. All these studies were carried out in high-income countries: the Netherlands [8,9,16], Australia [11] and Japan [12]. The **Table 1** summarizes the characteristics of the included studies, detailing both clinical and economic outcomes.

**Table 1:** Characteristics of studies included in the systematic review.

Author, Year	Country	Study design	Participants	Intervention	Number of participants	Age (years)	Clinical outcomes	Costs	Cost per outcome	ICER	Conclusions
Mori, 2021 [12]	Japan	Prospective	Women with unexplained infertility, PCOS, ovulation disorder, or mild male infertility undergoing non-ART treatment (including timing therapy, ovulation induction, or artificial insemination) for the first time	T: Three 30-minute sessions of education and care program within 6 months C: Did not receive program	T: 69 C: 104	<42 years T: 32.3±3.5 C: 32.7±3.7	*Pregnancy rate* T: 39.1% C: 32.7% *Mean time* *to pregnancy* T: 127 days C: 119 days	*Mean total* *cost* T: ¥96 390 ±107 558 C: ¥119 600 ±92 946	*Cost per pregnancy* T: ¥155 707 C: ¥304 589	NA	Education and care program required lower cost per patient to achieve pregnancy.
Oostingh, 2019 [9]	Nether lands	CEA	Women of subfertile couples who start their 1^st^ IVF cycle	T1: Mobile health (mHealth) coaching program Smarter Pregnancy (coaching on nutrition and lifestyle behaviors) for 24 weeks C: Usual care	793	18–45 years	*Pregnancy rate* T: 47% C: 36%	*Mean healthcare cost* T: €6 008 500 C: €6 214 800 *Mean societal cost* T: €7 492 400 C: €7 762 400	NA	*Health care perspective*: -€2250 per additional pregnancy *Societal perspective*: -€3050 per additional pregnancy	The mHealth coaching program Smarter Pregnancy is potentially cost saving.
Sim, 2013 [11,17]	Australia	RCT	Obese (BMI ≥ 30 kg/m^2^) women undertaking fertility treatment (i.e., IVF, ICSI, or subsequent cryostored embryo transfer cycles)	T: 12-week weight-loss intervention (i.e., very-low-energy diet, hypocaloric diet, multidisciplinary program, exercise, psychological/ behavioral advice) C: Control (i.e., recommendation for weight loss and printed material)	T: 27 C: 22	18–37 years T: 32.9±3.3 C: 32.8±3.1	*Pregnancy rate* T: 48% C: 14% *Live birth rate* T: 44% C: 14%	*Intervention cost* T: AU$12 457 C: AU$694	*Cost per pregnancy* T: AU$8422 C: AU$17 458	NA	Weight loss improved pregnancy rate and was cost-saving.
Steegers-Theunissen, 2020 [8]	Nether lands	CEA	Subfertile couples and obese women undergoing ART (i.e., IVF, ICSI, and IUI)	T1: Smarter Pregnancy (online tool) T2: LIFEstyle (outpatient and telephone consultation) T3: Smarter Pregnancy +LIFEstyle T4: Smoking cessation in men T5: Mindfulness program	NA	25–45 years	*Spontaneous pregnancy rate per year* T1: 13% T2: 10% T3: 13% T4: NA T5: NA *IVF*, *ICSI*, *IUI treatment rate per year* T1: -5, -3, -23 T2: -11, -19, -20 T3: -6, -5, -23 T4: -1, -0.4, NA T5: -12, -1, NA	NA	NA	*Cost saving on ART and pregnancy complication reduction per couple (healthcare perspective)* T1: €513 T2: €1163 T3: €586 T4: €41 T5: €36	Pre-conception lifestyle interventions are likely to be cost-effective.
Van Oers, 2017 [16]	Nether lands	CEA	Obese (BMI ≥ 29 kg/m^2^) infertile women	T: 6-month lifestyle intervention program preceding infertility treatment C: Prompt infertility treatment Infertility treatments include ovarian induction, IUI, IVF, ICSI	T: 280 C: 284	18–39 years T: 29.7 C: 29.8	*Healthy live birth rate* T: 27% C: 35%	*Mean total cost per woman* T: €4324 ±4276 C: €5603 ±4632	*Costs per* *healthy live birth within 24 months* T: €15 932 C: €15 912	*Hospital perspective* €15 845 per additional live birth rate	Lifestyle intervention is less effective and less costly as compared to prompt infertility treatment and therefore NOT cost-effective.

Abbreviations: AU$, Australian dollar; €, euro; ¥, Japanese yen; BMI, body mass index; C, control group; CEA, cost-effective analysis; ICER, incremental cost-effectiveness ratio (ICER = Δcost/Δeffect); ICSI, intracytoplasmic sperm injection; IUI, intrauterine insemination; IVF, *in vitro* fertilization; NA, not applicable; PCOS, polycystic ovary syndrome; T, treatment group.

### Details of the studied population

The **Table 1** presents the participant characteristics across each study. All studies focused on couples seeking or undergoing infertility treatments, such as ovarian induction, intrauterine insemination, or assisted reproductive technologies [8,9,11,12,16]. The inclusion criteria of the five studies capped the age of women between 37 and 45 years, with the mean ages of participants in the order of 30 to 33 years. The predominant cause of infertility across these studies was unexplained, although one study included women who were diagnosed with unexplained infertility, polycystic ovary syndrome, ovulation disorders, or mild male infertility [12]. Three studies specifically targeted women with obesity, indicated by a body mass index ≥ 29–30 kg/m^2^ [8,11,16]. The lifestyle interventions implemented comprised coaching programmes, psychological/behavioural counselling, dietary adjustments, physical activity encouragement, weight management, education via telephone and the internet, smoking cessation and mindfulness practices. The intervention durations ranged from 6 to 8 months. Control groups were given standard or immediate infertility care [9,11,12,16], except in one instance where five distinct lifestyle-related interventions were compared [8].

### Clinical outcomes

#### Pregnancy rate

A notable majority of the studies found that the intervention groups experienced higher pregnancy rates than did the control groups [9,11,12], with the rates ranging from 10% to 48% for the intervention groups and from 14% to 36% for the control groups. The intervention that yielded the most significant increase in pregnancy rates compared to those in the control group was a 12-week weight loss programme, featuring a very-low-energy diet, a hypocaloric diet, a multidisciplinary regimen, exercise and psychological/behavioural counselling. This intervention achieved a pregnancy rate of 48%, against 14% in the control group [11,17]. Moreover, in the Netherlands, an elevated pregnancy rate was observed in the group receiving a mobile health coaching programme, as opposed to the usual care group (47% vs. 36%, respectively) [9]. In Japan, a study by Mori et al., which assessed patients with various infertility causes, also revealed a greater pregnancy rate in participants receiving three 30-minute education and care sessions over 6 months than in the control group (39.1% vs. 32.7%, respectively, *P* > 0.05) [12]. Another study from the Netherlands reported annual spontaneous pregnancy rates of 13%, 13% and 10% for programs combining online with outpatient-based, online-based only and outpatient-based only interventions, respectively.

#### Live birth rate

The live birth rate was delineated in two studies [11,16]. In an Australian randomized controlled trial, 49 obese women facing infertility were randomly assigned to either a 12-week weight reduction programme or to receive only recommendations for weight loss alongside printed materials. This study revealed a notably higher healthy-live-birth rate in the intervention group than in the control group (44% vs. 14%, respectively) [11,17]. In contrast, research conducted in the Netherlands involving 577 obese women did not find that a 6-month lifestyle intervention programme, undertaken prior to commencing infertility treatment, increased live birth rates when juxtaposed with the control group receiving immediate treatment (27% vs. 35%, respectively, *P* < 0.05) [16].

### Economic outcomes

#### Total cost

The calculation and reporting of the total costs for the intervention and control groups varied across the five studies, leading to differing cost amounts. The majority of the studies estimated costs from a hospital or healthcare provider perspective [8,9,16], encompassing all direct medical costs associated with lifestyle interventions, infertility treatments and other pertinent healthcare utilization costs during the treatment process (e.g., medication, pregnancy and complications). Notably, Oostingh et al. [9] also included costs from a societal perspective, which comprised all healthcare costs plus indirect costs outside the healthcare setting, such as work absence. This approach revealed that among 793 patients, the mean societal costs were approximately €1 500 000 higher than those calculated from the healthcare perspective. Mori et al. [12] evaluated economic outcomes based on medical expenses either paid by patients or covered by public insurance. Another study was a randomized controlled trial [11,17], factoring in administration and material costs for implementing the 12-week multidisciplinary weight loss programme and the costs associated with assisted reproductive procedures. Intriguingly, the majority of studies that reported total costs found that the costs for control groups were greater than those for lifestyle intervention groups [9,12,16].

#### Cost per outcome

Two studies documented the cost per pregnancy, revealing that the cost in the control groups was approximately twice as high as that in the intervention groups [11,12]. Furthermore, the cost per live birth within a 24-month timeframe was reported in one study [16]. This study revealed comparable costs between the group undergoing lifestyle intervention preceding infertility treatment and the group receiving immediate infertility treatment.

#### Cost-effectiveness

Three out of five studies addressed the cost-effectiveness of lifestyle interventions relative to the absence of such interventions [8,9,16]. Two of these studies, both from the Netherlands, found that lifestyle intervention programmes were cost-saving compared to standard care [8,9]. These analyses assessed the cost-effectiveness of a range of lifestyle interventions, including online-based, mobile-based, telephone-based and outpatient-based interventions, with one study [8] additionally evaluating smoking cessation and mindfulness programs.

The first study [9] developed an economic model utilizing data from 793 infertile women who were undergoing their first cycle of *in vitro* fertilization treatment. This model illustrated that the lifestyle modification programme resulted in cost savings of €2250 per additional pregnancy from a healthcare perspective and €3050 from a societal perspective, which calculated using the total costs that included both healthcare-related costs and costs incurring outside the healthcare setting, such as costs due to absence at work). A second model-based study [8] quantified the financial savings per couple resulting from various lifestyle interventions, highlighting differing cost-benefit outcomes across the types of interventions. The outpatient with telephone consultation lifestyle intervention (LIFEstyle), aimed at obese women seeking infertility treatment, yielded the highest cost-benefit of €1163 per couple for assisted reproductive technologies and pregnancy complications. The LIFEstyle programme surpassed other interventions such as an online coaching programme (Smarter Pregnancy; €513), a combined Smarter Pregnancy and LIFEstyle intervention (€586), a smoking-cessation-in-men programme (€41) and a mindfulness programme (€36). However, when considering the size of the target groups, the combined intervention emerged as the most cost-saving intervention on an annual basis (€27 million; total target group = 46 000). It was followed by Smarter Pregnancy (€24 million; total annual target group = 46 000), LIFEstyle (€6 million; total target group = 5400), the mindfulness programme (€5 million; total annual target group = 13 700) and smoking cessation in men (€0.1 million; total annual target group = 3200).

Conversely, one study [16] deemed that the lifestyle intervention programme was not cost-effective. This research, which was also undertaken in the Netherlands, focused on 577 obese women with infertility. Participants were divided into two groups: one group received a 6-month lifestyle intervention before starting infertility treatment (n = 280), while the other group underwent immediate infertility treatment (n = 284). Although the total cost for the intervention group was lower than that for the control group, the former achieved a lower healthy live birth rate. Consequently, the incremental cost-effectiveness ratio was derived using the cost and clinical effect of the intervention group as benchmarks. The study concluded that with an incremental cost-effectiveness ratio of €15 845 per additional live birth from the hospital perspective, lifestyle intervention was not considered cost-effective.

#### Sensitivity analysis

In assessing the robustness of their findings, three cost-effectiveness studies [8,9,16] undertook deterministic and/or probabilistic sensitivity analyses. These analyses aimed to evaluate the impact of uncertain model input parameters on the model outputs. Oostingh et al. established that the mobile health (mHealth) programme was predominantly regarded as cost-saving. Nonetheless, the incremental cost-effectiveness ratios tended to increase in scenarios where the intervention’s effectiveness was compromised by reduced compliance and lower ongoing pregnancy rates [9]. Steegers-Theunissen et al. explored the range of cost savings each intervention could yield, comparing the least and most favourable scenario calculations [8]. Among the five interventions examined, only smoking cessation and mindfulness programmes showed a potential shift from delivering cost benefits to yielding no additional cost benefits in the least favourable scenarios. Van Oers et al.’s study [16] demonstrated considerable robustness through bootstrap analysis, with 98% of the 5000 replications indicating that the lifestyle intervention preceding infertility treatment would likely incur lower costs but also a lower rate of healthy singleton births at term within 24 months, as opposed to prompt infertility treatment. Conversely, the remaining 2% of replications suggested that the intervention group could achieve additional live births at a reduced cost compared with the immediate treatment approach.

## Discussion

Our findings illuminate the effectiveness and cost-effectiveness of incorporating lifestyle interventions in the treatment of infertility. The present evidence underscores the substantial economic burden associated with conventional infertility treatments, including medication, intrauterine insemination and assisted reproductive technologies [10]. In contrast, our study revealed that the integration of lifestyle interventions into the infertility treatment paradigm predominantly results in cost-saving outcomes.

The efficacy of lifestyle interventions as a treatment for infertility has been corroborated, positioning them among several effective strategies to combat infertility. The influence of daily life activities on fertility outcomes underpins the clinical benefit of such interventions, which can be administered through various means, including dietary recommendations, exercise and weight loss programmes, smoking cessation, motivational and psychological consultations, behavioural advice and educative initiatives. Specifically, adherence to a Mediterranean diet has been associated with a 40% increase in pregnancy rates among couples undergoing *in vitro* fertilization or intracytoplasmic sperm injection procedures [7].

Moreover, alcohol consumption has been implicated in fertility issues due to its potential to elevate oestrogen levels, consequently reducing follicle-stimulating hormone levels and ovulation. Nonetheless, the link between alcohol consumption and infertility remains inconclusive in some studies [3,7]. A prospective cohort study in Denmark found that females who consumed more than 13 servings of alcohol weekly experienced reduced fertility [18]. Similarly, caffeine intake is known to elevate oestrogen levels. This hinders ovulation and affects corpus luteal function, resulting in the time to conceive being extended [7]. The association between caffeine intake and fertility was observed in a study tracking 3628 women attempting to conceive, where a 4-year follow-up showed that the daily consumption of more than two servings of caffeine adversely impacted fertility [19]. Thus, advising patients to moderate their alcohol and caffeine intake may enhance fertility prospects. Among the five studies reviewed, four incorporated dietary/nutritional recommendations within their lifestyle interventions [8,9,11,16] and three addressed alcohol intake [8,9,11]. However, none of the five studies explicitly focused on caffeine consumption.

Encouraging an optimal amount of exercise and physical activity, in addition to weight reduction, is crucial in managing infertility among women with obesity [7]. Obesity is linked to hyperinsulinemia and an increased conversion of androgens to oestrogens through aromatization, leading to elevated free testosterone and oestrogen levels. These hormonal imbalances are known to impair folliculogenesis and promote follicular atresia due to increased luteinizing hormone levels and androgen-to-oestrogen ratios [20]. A North American study enrolling 2062 individuals planning pregnancies investigated the association between body mass index and infertility. The study identified a negative correlation between an increase in body mass index and the fecundability ratio [21]. Among the three studies that assessed the cost-effectiveness of lifestyle interventions in obese women with infertility, two [8,11] found that weight loss programmes were cost-saving or cost-effective. The third study [16] noted lower costs in the group receiving lifestyle interventions before infertility treatment than in the group receiving immediate infertility treatment. However, the third study did not show increased effectiveness in terms of the vaginal birth rate of healthy singletons at term within a 24-month follow-up period.

Smoking cessation is a vital component that should be incorporated into lifestyle interventions for managing infertility, as underscored in three of the studies included in this review [8,9,12]. Smoking adversely impacts the follicular microenvironment through mechanisms yet to be fully elucidated [22]. A study examining 3773 Danish individuals planning pregnancies investigated the relationship between smoking and fertility. The analysis revealed that both current and former regular smokers with significant cumulative exposure to cigarette smoke experienced delays in conception compared to nonsmokers [23].

Our systematic review represents an inaugural study addressing the economic outcomes associated with lifestyle interventions for managing infertility, thereby filling a notable gap in the literature. By conducting an exhaustive and systematic search across three validated medical databases, we aimed to encapsulate both the clinical benefits and economic aspects of lifestyle interventions. The findings from our study suggest that, predominantly, additional lifestyle interventions not only offer cost-saving advantages but also yield cost-beneficial outcomes for patients undergoing infertility treatments, as opposed to those who do not partake in any lifestyle intervention programme. Importantly, four out of the five studies reviewed [8,9,11,12] underscore the cost-effectiveness of such lifestyle intervention programmes. In contrast, only a single study posited that lifestyle modification for obese women with infertility was not cost-effective [16]. This disparity might be attributed to the study’s relatively brief 24-month follow-up period and its focus on the clinical outcome of the vaginal birth rate of healthy singletons at term. The study authors mentioned that lifestyle interventions preceding infertility treatments might exhibit greater cost-effectiveness when evaluated over longer follow-up periods and with the inclusion of broader endpoints in the analysis.

According to our findings, it is notable that the total costs, intervention costs, costs per outcome as well as incremental cost-effectiveness ratios are quite varied among studies, even when they were converted into the same currency unit. These can be explained by various reasons including characteristics of the provided lifestyle interventions, effectiveness of the interventions, costs of baseline treatments, economic model assumptions and analysis perspectives, etc. It is crucial to remark that a direct comparison of economic outcomes across the included studies might not be suitable to perform. The main reasons are not only that the studies themselves had differences in eligibility criteria and there were varieties of the lifestyle interventions conducted in the studies, but each country or setting also has different economic context.

However, when focusing only on the intervention costs, there was one study that evaluated the cost-effectiveness of more than one type of lifestyle intervention [8]. It seemed that the online-based intervention (including mobile apps and web-based tools) required lower cost than did the outpatient-based intervention (€120 versus €246 per 2 years of interventions, respectively) [8]. Nonetheless, regarded to the same study [8], the outpatient-based intervention resulted in more cost saving per couple than the online-based intervention as it was provided for a specific obese female population who might gained more benefit from the intervention, compared to other infertile populations. The smoking cessation progremme that was implemented in smoking male population resulted in the least cost-saving amount compared to other types of interventions. These findings indicated that even if the integration of lifestyle interventions seemed to be cost-effective, as compared to conventional care alone, the magnitude of cost-saving also depended on the target population. In the real-world situation, the policymakers are suggested to prioritize the treatments for the population who potentially gain more benefit from the intervention. Additionally, the interpretations in economic aspects and application of these outcomes depended on multiple factors, such as the willingness-to-pay threshold and availability of healthcare resources. Moreover, the process of evidence-based policy development and implementation are conditional on national prioritization which leads to differences in budget allocation.

Our systematic review is subject to limitations that warrant consideration. Firstly, the inclusion of a limited number of studies sourced from three databases might impact the accuracy and comprehensiveness of our findings. Secondly, all included studies originated in high-income countries and were mostly conducted in female participants, potentially limiting the generalizability of the results. The applicability of our results in low- and middle-income contexts must be approached with caution due to distinct disparities in factors such as treatment costs, healthcare resources, patient health literacy and self-care capabilities across different income settings. The application in other high-income countries where the reimbursement and health insurance policy are different from the included studies, should, as well, be proceeded with cuation. Thirdly, the predominant reliance on pregnancy rate as an endpoint in the majority of included studies may affect the interpretability of our results, especially when study conditions and model assumptions vary. Furthermore, a comprehensive economic evaluation should consider additional outcomes affecting the cost of treatment, including time-to-pregnancy, pregnancy complications, miscarriage rates and healthy live birth metrics, as well as how participant characteristics and the specifics of lifestyle interventions influence both cost and clinical outcomes. Consequently, the findings of this systematic review should be interpreted with due caution.

## Conclusion

In conclusion, our systematic review contributes significantly to bridging the knowledge gap regarding the economic implications of incorporating lifestyle interventions into infertility treatments. These interventions include coaching programmes, educational initiatives, psychological/behavioural counselling, dietary adjustments, physical activity, weight management, smoking cessation and mindfulness programmes for couples undergoing infertility treatments. They have shown promising clinical enhancements, specifically, increasing pregnancy and live birth rates. Importantly, most of the studies indicated that these lifestyle interventions were cost-effective compared with control groups not receiving such interventions. However, all of the studies were conducted within the context of high-income countries, and mostly focused on the lifestyle interventions for female infertile patients. More cost-effectiveness studies of lifestyle interventions for male infertile patients are still in need.

## Supporting information

S1 File PRISMA checklist. (PDF)

S2 File Search strategies. (PDF)

S3 File Quality assessment of included studies. (PDF)
