# Supplementary material for: Economic evaluation of lifestyle interventions in infertility management: A systematic review
Source: PLoS One. 2024 Aug 23;19(8):e0306419. doi: 10.1371/journal.pone.0306419 (PMC11343367; doi:10.1371/journal.pone.0306419)
Supplement: S2 File — (PDF) [file pone.0306419.s002.pdf]

Supplementary data 2: search strategies

Embase (n = 5712)

1. 'infertility'/exp OR 'infertility'
2. 'subfertility'/exp OR 'subfertility'
3. #1 OR #2
4. 'economic evaluation'/exp OR 'economic evaluation'
5. 'cost effective' AND ('analysis'/exp OR analysis)
6. 'cea'/exp OR cea
7. 'cost benefit analysis'/exp OR 'cost benefit analysis'
8. cua
9. cba
10. 'cost utility analysis'/exp OR 'cost utility analysis'
11. 'cost minimization analysis'/exp OR 'cost minimization analysis'
12. cma
13. 'cost'/exp OR 'cost'
14. 'ovary hyperstimulation'/exp OR 'ovary hyperstimulation'
15. 'fertility promoting agent'/exp OR 'fertility promoting agent'
16. ('fertility'/exp OR fertility) AND ('drug'/exp OR drug)
17. 'clomifene citrate'/exp OR 'clomifene citrate'
18. 'selective estrogen receptor modulator'/exp OR 'selective estrogen receptor modulator'
19. cc
20. 'serm'/exp OR serm
21. 'letrozole'/exp OR 'letrozole'
22. 'non steroidal' AND ('inhibitor'/exp OR inhibitor) AND of AND ('aromatase'/exp OR aromatase)
23. 'aromatase inhibitor'/exp OR 'aromatase inhibitor'
24. 'metformin'/exp OR 'metformin'
25. mfm
26. 'bromocriptine'/exp OR 'bromocriptine'
27. 'dopamine receptor stimulating agent'/exp OR 'dopamine receptor stimulating agent'
28. 'bromocriptine mesilate'/exp OR 'bromocriptine mesilate'
29. bromocryptin
30. 'chorionic gonadotropin'/exp OR 'chorionic gonadotropin'
31. 'choriogonadotropin alfa (drug)'/exp OR 'choriogonadotropin alfa (drug)'
32. 'gonadotropin'/exp OR 'gonadotropin'
33. 'superovulation'/exp OR 'superovulation'
34. gn
35. 'follitropin'/exp OR 'follitropin'
36. 'lh'/exp OR lh
37. 'luteinizing hormone'/exp OR 'luteinizing hormone'
38. lutrophin
39. 'follitropin'/exp OR 'follitropin'
40. 'in vitro fertilization'/exp OR 'in vitro fertilization'
41. 'ivf'/exp OR 'ivf'
42. invitro AND ('fertilization'/exp OR fertilization)

43. invitro AND fertilization
44. 'intracytoplasmic sperm injection'/exp OR 'intracytoplasmic sperm injection'
45. 'art'/exp OR art
46. 'infertility therapy'/exp OR 'infertility therapy'
47. 'assisted reproduction'/exp OR 'assisted reproduction'
48. 'intrauterine insemination'/exp OR 'intrauterine insemination'
49. iui
50. 'intra uterine' AND ('insemination'/exp OR insemination)
51. 'counseling'/exp OR 'counseling'
52. 'e-counseling'/exp OR 'e-counseling'
53. 'lifestyle'/exp OR 'lifestyle'
54. 'attitude to health'/exp OR 'attitude to health'
55. 'behavior therapy'/exp OR 'behavior therapy'
56. 'decision support system'/exp OR 'decision support system'
57. 'patient education'/exp OR 'patient education'
58. 'drinking behavior'/exp OR 'drinking behavior'
59. 'health behavior'/exp OR 'health behavior'
60. 'smoking'/exp OR 'smoking'
61. 'cannabis'/exp OR 'cannabis'
62. 'reproductive behavior'/exp OR 'reproductive behavior'
63. 'tobacco use'/exp OR 'tobacco use'
64. ('tobacco'/exp OR tobacco) AND ('reduction'/exp OR reduction)
65. 'street drug'/exp OR 'street drug'
66. 'drug abuse'/exp OR 'drug abuse'
67. 'recreational drug'/exp OR 'recreational drug'
68. 'cocaine'/exp OR 'cocaine'
69. ('tobacco'/exp OR tobacco) AND ('cessation'/exp OR cessation)
70. 'alcohol'/exp OR 'alcohol'
71. 'caffeine'/exp OR 'caffeine'
72. 'coffee'/exp OR 'coffee'
73. 'low calorie diet'/exp OR 'low calorie diet'
74. 'diet restriction'/exp OR 'diet restriction'
75. 'low carbohydrate diet'/exp OR 'low carbohydrate diet'
76. 'diet supplementation'/exp OR 'diet supplementation'
77. 'healthy diet'/exp OR 'healthy diet'
78. 'diet therapy'/exp OR 'diet therapy'
79. 'low fat diet'/exp OR 'low fat diet'
80. 'nutraceutical'/exp OR 'nutraceutical'
81. 'vitamin'/exp OR 'vitamin'
82. folic AND ('acid'/exp OR acid)
83. 'iodine'/exp OR 'iodine'
84. 'weight'/exp OR 'weight'
85. 'obesity'/exp OR 'obesity'
86. obese
87. 'body mass'/exp OR 'body mass'
88. 'bmi'/exp OR 'bmi'

89. 'exercise'/exp OR 'exercise'
90. 'physical activity'/exp OR 'physical activity'
91. 'sexual intercourse'/exp OR 'sexual intercourse'
92. 'timing'/exp OR 'timing'
93. 'frequency'/exp OR 'frequency'
94. 'compliance'/exp OR compliance
95. #92 OR #93 OR #94
96. #91 AND #95
97. 'yoga'/exp OR 'yoga'
98. 'jogging'/exp OR 'jogging'
99. 'hypnosis'/exp OR 'hypnosis'
100. 'meditation'/exp OR 'meditation'
101. 'walking'/exp OR walking
102. 'body weight loss'/exp OR 'body weight loss'
103. ai
104. #14 OR #15 OR #16 OR #17 OR #18 OR #19 OR #20 OR #21 OR #22 OR #23 OR #24 OR #25 OR #26 OR #27 OR #28 OR #29 OR #30 OR #31 OR #32 OR #33 OR #34 OR #35 OR #36 OR #37 OR #38 OR #39 OR #40 OR #41 OR #42 OR #43 OR #44 OR #45 OR #46 OR #47 OR #48 OR #49 OR #50 OR #51 OR #52 OR #53 OR #54 OR #55 OR #56 OR #57 OR #58 OR #59 OR #60 OR #61 OR #62 OR #63 OR #64 OR #65 OR #66 OR #67 OR #68 OR #69 OR #70 OR #71 OR #72 OR #73 OR #74 OR #75 OR #76 OR #77 OR #78 OR #79 OR #80 OR #81 OR #82 OR #83 OR #84 OR #85 OR #86 OR #87 OR #88 OR #89 OR #90 OR #91 OR #92 OR #93 OR #94 OR #95 OR #96 OR #97 OR #98 OR #99 OR #100 OR #101 OR #102 OR #103
105. #3 AND #104
106. #4 OR #5 OR #6 OR #7 OR #8 OR #9 OR #10 OR #11 OR #12 OR #13
107. #105 AND #106

Pubmed (n = 481)

1. (infertili*) AND (infertili*[MeSH Terms])
2. subfertili*
3. ((infertili*) AND (infertili*[MeSH Terms])) OR (subfertili*)
4. (Clomiphene citrate) AND (Clomiphene citrate[MeSH Terms])
5. (cc) OR (serm)
6. (clomid) OR (clomid[MeSH Terms])
7. (selective estrogen receptor modulator) AND (selective estrogen receptor modulator[MeSH Terms])
8. (letrozole) AND (letrozole[MeSH Terms])
9. (CGS 20267) OR (CGS 20267[MeSH Terms])
10. (non-steroidal inhibitor of aromatase) AND (non-steroidal inhibitor of aromatase[MeSH Terms])
10. (aromatase inhibitor) AND (aromatase inhibitor[MeSH Terms])
11. AI
12. (Femara) AND (Femara[MeSH Terms])
13. (metformin) AND (metformin[MeSH Terms])
14. mfm

15. Fortamet
16. (ovarian stimulation) AND (ovarian stimulation[MeSH Terms])
17. ovarian hyperstimulation
18. (fertility agent) AND (fertility agent[MeSH Terms])
19. fertility drug
20. (induction of ovulation) AND (induction of ovulation[MeSH Terms])
21. (gonadotropin-releasing hormone) AND (gonadotropin-releasing hormone[MeSH Terms])
22. (glucophage) AND (glucophage[MeSH Terms])
23. (biomet) AND (biomet[MeSH Terms])
24. riomet
25. (Bromocriptine) AND (Bromocriptine[MeSH Terms])
26. (dopamine agonist) AND (dopamine agonist[MeSH Terms])
27. (Parlodel) AND (Parlodel[MeSH Terms])
28. cycloset
29. (CB-154) AND (CB-154[MeSH Terms])
30. cb 154
31. (Bromocryptin) AND (Bromocryptin[MeSH Terms])
32. (Human chorionic gonadotropin) AND (Human chorionic gonadotropin[MeSH Terms])
33. hCG
34. Novarel
35. Ovidrel
36. (Pregnyl) AND (Pregnyl[MeSH Terms])
37. Profasi
38. Follutein
39. (Gonadotropin) AND (Gonadotropin[MeSH Terms])
40. (Gonadotrophin) AND (Gonadotrophin[MeSH Terms])
41. (superovulation) AND (superovulation[MeSH Terms])
42. ((gn) OR (LH)) OR (FSH)
43. (Luteinizing hormone) AND (Luteinizing hormone[MeSH Terms])
44. (luteinising hormone) AND (luteinising hormone[MeSH Terms])
45. (lutropin) AND (lutropin[MeSH Terms])
46. (Follicle-stimulating hormone) AND (Follicle-stimulating hormone[MeSH Terms])
47. (follitropin) AND (follitropin[MeSH Terms])
48. (Fertilization in Vitro) AND (Fertilization in Vitro[MeSH Terms])
49. ivf
50. (invitro fertilization) AND (invitro fertilization[MeSH Terms])
51. (in vitro fertilization) AND (in vitro fertilization[MeSH Terms])
52. (in-vitro fertilization) AND (in-vitro fertilization[MeSH Terms])
53. (invitro fertilisation) AND (invitro fertilisation[MeSH Terms])
54. (in vitro fertilisation) AND (in vitro fertilisation[MeSH Terms])
55. (in-vitro fertilisation) AND (in-vitro fertilisation[MeSH Terms])
56. (Sperm Injection) AND (Sperm Injection[MeSH Terms])
57. ICSI
58. ART

59. (assisted reproductive technology) AND (assisted reproductive technology[MeSH Terms])
60. (Assisted reproduction) AND (Assisted reproduction[MeSH Terms])
61. (Intrauterine insemination) AND (Intrauterine insemination[MeSH Terms])
62. IUI
63. (Intra-uterine insemination) AND (Intra-uterine insemination[MeSH Terms])
64. (counseling) AND (counseling[MeSH Terms])
65. (e-counseling) AND (e-counseling[MeSH Terms])
66. (lifestyle) AND (lifestyle[MeSH Terms])
67. (life style) AND (life style[MeSH Terms])
68. (attitude to health) AND (attitude to health[MeSH Terms])
69. (behavior therapy) AND (behavior therapy[MeSH Terms])
70. decision support
71. (patient education) AND (patient education[MeSH Terms])
72. (drinking behavior) AND (drinking behavior[MeSH Terms])
73. (health behavior) AND (health behavior[MeSH Terms])
74. (smoking) AND (smoking[MeSH Terms])
75. (cannabis) AND (cannabis[MeSH Terms])
76. (reproductive behavior) AND (reproductive behavior[MeSH Terms])
77. (tobacco use) AND (tobacco use[MeSH Terms])
78. tobacco reduction
79. (street drug) AND (street drug[MeSH Terms])
80. (drug abuse) AND (drug abuse[MeSH Terms])
81. (recreational drug) AND (recreational drug[MeSH Terms])
82. (cocaine) AND (cocaine[MeSH Terms])
83. (marijuana) AND (marijuana[MeSH Terms])
84. (tobacco cessation) AND (tobacco cessation[MeSH Terms])
85. (alcohol) AND (alcohol[MeSH Terms])
86. (coffee) AND (coffee[MeSH Terms])
87. (low calory diet) AND (low calory diet[MeSH Terms])
88. diet restriction
89. (low carbohydrate diet) AND (low carbohydrate diet[MeSH Terms])
90. diet supplementation
91. (healthy diet) AND (healthy diet[MeSH Terms])
92. (diet therapy) AND (diet therapy[MeSH Terms])
93. (low fat diet) AND (low fat diet[MeSH Terms])
94. (nutraceutical) AND (nutraceutical[MeSH Terms])
95. (vitamin) AND (vitamin[MeSH Terms])
96. (folic acid) AND (folic acid[MeSH Terms])
97. (iodine) AND (iodine[MeSH Terms])
98. (weight) AND (weight[MeSH Terms])
99. (obes*) AND (obes*[MeSH Terms])
100. (overweight) AND (overweight[MeSH Terms])
101. (Body mass index) AND (Body mass index[MeSH Terms])
102. BMI
103. (exercis*) AND (exercis*[MeSH Terms])

104. (physical activit*)
105. (intercourse) AND (intercourse[MeSH Terms])
106. (frequency) AND (frequency[MeSH Terms])
107. (compliance) AND (compliance[MeSH Terms])
108. timing
109. (((frequency) AND (frequency[MeSH Terms])) OR ((compliance) AND (compliance[MeSH Terms]))) OR (timing)
110. ((((frequency) AND (frequency[MeSH Terms])) OR ((compliance) AND (compliance[MeSH Terms]))) OR (timing)) AND ((intercourse) AND (intercourse[MeSH Terms]))
111. (yoga) AND (yoga[MeSH Terms])
112. (jogging) AND (jogging[MeSH Terms])
113. (hypnosis) AND (hypnosis[MeSH Terms])
114. (meditation) AND (meditation[MeSH Terms])
115. (walking) AND (walking[MeSH Terms])
116. (folate) AND (folate[MeSH Terms])
117. (ethanol) AND (ethanol[MeSH Terms])
118. (body weight loss) AND (body weight loss[MeSH Terms])
119. (economic evaluation) AND (economic evaluation[MeSH Terms])
120. (Cost-effective analysis) OR (cea)
121. (Cost minimization analysis) AND (Cost minimization analysis[MeSH Terms])
122. ((cma) OR (cba)) OR (cua)
123. (Cost utility analysis) AND (Cost utility analysis[MeSH Terms])
124. (Cost benefit analysis) AND (Cost benefit analysis[MeSH Terms])
125. (cost) AND (cost[MeSH Terms])
126. ((((((((economic evaluation) AND (economic evaluation[MeSH Terms])) OR ((Cost benefit analysis) AND (Cost benefit analysis[MeSH Terms]))) OR ((Cost utility analysis) AND (Cost utility analysis[MeSH Terms]))) OR (cua)) OR (cba)) OR (cma)) OR ((Cost minimization analysis) AND (Cost minimization analysis[MeSH Terms]))) OR ((Cost-effective analysis) OR (cea))) OR ((cost) AND (cost[MeSH Terms]))
127. (((((((((((((((((((((gonadotropin-releasing hormone) AND (gonadotropin-releasing hormone[MeSH Terms])) OR ((induction of ovulation) AND (induction of ovulation[MeSH Terms]))) OR (fertility drug)) OR ((fertility agent) AND (fertility agent[MeSH Terms]))) OR (ovarian hyperstimulation)) OR ((ovarian stimulation) AND (ovarian stimulation[MeSH Terms]))) OR (Fortamet)) OR (mfm)) OR ((metformin) AND (metformin[MeSH Terms]))) OR ((Femara) AND (Femara[MeSH Terms]))) OR (AI)) OR ((aromatase inhibitor) AND (aromatase inhibitor[MeSH Terms]))) OR ((non-steroidal inhibitor of aromatase) AND (non-steroidal inhibitor of aromatase[MeSH Terms]))) OR ((CGS 20267) OR (CGS 20267[MeSH Terms]))) OR ((letrozole) AND (letrozole[MeSH Terms]))) OR ((selective estrogen receptor modulator) AND (selective estrogen receptor modulator[MeSH Terms]))) OR ((clomid) OR (clomid[MeSH Terms]))) OR ((cc) OR (serm))) OR ((Clomifene citrate) AND (Clomifene citrate[MeSH Terms]))) OR
(((glucophage) AND (glucophage[MeSH Terms])) OR ((biomet) AND (biomet[MeSH Terms]))) OR ((body weight loss) AND (body weight loss[MeSH Terms]))) OR ((ethanol) AND (ethanol[MeSH Terms]))) OR ((folate) AND (folate[MeSH Terms]))) OR ((walking) AND (walking[MeSH

Terms]))) OR ((meditation) AND (meditation[MeSH Terms]))) OR ((hypnosis) AND (hypnosis[MeSH Terms]))) OR ((jogging) AND (jogging[MeSH Terms]))) OR ((yoga) AND (yoga[MeSH Terms]))) OR (((((frequency) AND (frequency[MeSH Terms])) OR ((compliance) AND (compliance[MeSH Terms]))) OR (timing)) AND ((intercourse) AND (intercourse[MeSH Terms])))) OR ((physical activit*)) OR ((exercis*) AND (exercis*[MeSH Terms]))) OR (BMI)) OR ((Body mass index) AND (Body mass index[MeSH Terms]))) OR ((overweight) AND (overweight[MeSH Terms]))) OR ((obes*) AND (obes*[MeSH Terms]))) OR ((weight) AND (weight[MeSH Terms]))) OR ((iodine) AND (iodine[MeSH Terms]))) OR ((folic acid) AND (folic acid[MeSH Terms]))) OR ((vitamin) AND (vitamin[MeSH Terms]))) OR ((nutraceutical) AND (nutraceutical[MeSH Terms]))) OR ((low fat diet) AND (low fat diet[MeSH Terms]))) OR ((diet therapy) AND (diet therapy[MeSH Terms]))) OR ((healthy diet) AND (healthy diet[MeSH Terms]))) OR (diet supplementation)) OR ((low carbohydrate diet) AND (low carbohydrate diet[MeSH Terms]))) OR (diet restriction)) OR ((low calory diet) AND (low calory diet[MeSH Terms]))) OR ((coffee) AND (coffee[MeSH Terms]))) OR ((caffeine) AND (caffeine[MeSH Terms]))) OR ((alcohol) AND (alcohol[MeSH Terms]))) OR ((tobacco cessation) AND (tobacco cessation[MeSH Terms]))) OR ((marijuana) AND (marijuana[MeSH Terms]))) OR ((cocaine) AND (cocaine[MeSH Terms]))) OR ((recreational drug) AND (recreational drug[MeSH Terms]))) OR ((drug abuse) AND (drug abuse[MeSH Terms]))) OR ((street drug) AND (street drug[MeSH Terms]))) OR (tobacco reduction)) OR ((tobacco use) AND (tobacco use[MeSH Terms]))) OR ((reproductive behavior) AND (reproductive behavior[MeSH Terms]))) OR ((cannabis) AND (cannabis[MeSH Terms]))) OR ((smoking) AND (smoking[MeSH Terms]))) OR ((health behavior) AND (health behavior[MeSH Terms]))) OR ((drinking behavior) AND (drinking behavior[MeSH Terms]))) OR ((patient education) AND (patient education[MeSH Terms]))) OR (decision support)) OR ((behavior therapy) AND (behavior therapy[MeSH Terms]))) OR ((attitude to health) AND (attitude to health[MeSH Terms]))) OR ((life style) AND (life style[MeSH Terms]))) OR ((lifestyle) AND (lifestyle[MeSH Terms]))) OR ((e-counseling) AND (e-counseling[MeSH Terms]))) OR ((counseling) AND (counseling[MeSH Terms]))) OR ((Intra-uterine insemination) AND (Intra-uterine insemination[MeSH Terms]))) OR (IUI)) OR ((Intrauterine insemination) AND (Intrauterine insemination[MeSH Terms]))) OR ((Assisted reproduction) AND (Assisted reproduction[MeSH Terms]))) OR ((assisted reproductive technology) AND (assisted reproductive technology[MeSH Terms]))) OR (ART)) OR (ICSI)) OR ((Sperm Injection) AND (Sperm Injection[MeSH Terms]))) OR ((in-vitro fertilisation) AND (in-vitro fertilisation[MeSH Terms]))) OR ((in vitro fertilisation) AND (in vitro fertilisation[MeSH Terms]))) OR ((invitro fertilisation) AND (invitro fertilisation[MeSH Terms]))) OR ((in-vitro fertilization) AND (in-vitro fertilization[MeSH Terms]))) OR ((in vitro fertilization) AND (in vitro fertilization[MeSH Terms]))) OR ((invitro fertilization) AND (invitro fertilization[MeSH Terms]))) OR (ivf)) OR ((Fertilization in Vitro) AND (Fertilization in Vitro[MeSH Terms]))) OR ((follitropin) AND (follitropin[MeSH Terms]))) OR ((Follicle-stimulating hormone) AND (Follicle-stimulating hormone[MeSH Terms]))) OR ((lutropin) AND (lutropin[MeSH Terms]))) OR ((luteinising hormone) AND (luteinising hormone[MeSH Terms]))) OR ((Luteinizing hormone) AND (Luteinizing hormone[MeSH Terms]))) OR (((gn) OR (LH)) OR (FSH))) OR ((superovulation) AND (superovulation[MeSH Terms]))) OR ((Gonadotrophin) AND (Gonadotrophin[MeSH Terms]))) OR ((Gonadotropin) AND (Gonadotropin[MeSH Terms]))) OR (Follutein)) OR

(Profasi)) OR ((Pregnyl) AND (Pregnyl[MeSH Terms]))) OR (Ovidrel)) OR (Novarel)) OR ((Human chorionic gonadotrophin) AND (Human chorionic gonadotrophin[MeSH Terms]))) OR (hCG)) OR ((Human chorionic gonadotropin) AND (Human chorionic gonadotropin[MeSH Terms]))) OR ((Bromocryptin) AND (Bromocryptin[MeSH Terms]))) OR (cb 154)) OR ((CB-154) AND (CB-154[MeSH Terms]))) OR (cycloset)) OR ((Parlodel) AND (Parlodel[MeSH Terms]))) OR ((dopamine agonist) AND (dopamine agonist[MeSH Terms]))) OR ((Bromocriptine) AND (Bromocriptine[MeSH Terms]))) OR (riomet)) OR ((biomet) AND (biomet[MeSH Terms]))))) AND (((infertili*) AND (infertili*[MeSH Terms])) OR (subfertili*))

128. ((((((((((economic evaluation) AND (economic evaluation[MeSH Terms])) OR ((Cost benefit analysis) AND (Cost benefit analysis[MeSH Terms]))) OR ((Cost utility analysis) AND (Cost utility analysis[MeSH Terms]))) OR (cua)) OR (cba)) OR (cma)) OR ((Cost minimization analysis) AND (Cost minimization analysis[MeSH Terms]))) OR ((Cost-effective analysis) OR (cea))) OR ((cost) AND (cost[MeSH Terms]))) AND ((((((((((((((((((((((gonadotropin-releasing hormone) AND (gonadotropin-releasing hormone[MeSH Terms])) OR ((induction of ovulation) AND (induction of ovulation[MeSH Terms]))) OR (fertility drug)) OR ((fertility agent) AND (fertility agent[MeSH Terms]))) OR (ovarian hyperstimulation)) OR ((ovarian stimulation) AND (ovarian stimulation[MeSH Terms]))) OR (Fortamet)) OR (mfm)) OR ((metformin) AND (metformin[MeSH Terms]))) OR ((Femara) AND (Femara[MeSH Terms]))) OR (AI)) OR ((aromatase inhibitor) AND (aromatase inhibitor[MeSH Terms]))) OR ((non-steroidal inhibitor of aromatase) AND (non-steroidal inhibitor of aromatase[MeSH Terms]))) OR ((CGS 20267) OR (CGS 20267[MeSH Terms]))) OR ((letrozole) AND (letrozole[MeSH Terms]))) OR ((selective estrogen receptor modulator) AND (selective estrogen receptor modulator[MeSH Terms]))) OR ((clomid) OR (clomid[MeSH Terms]))) OR ((cc) OR (serm))) OR ((Clomifene citrate) AND (Clomifene citrate[MeSH Terms]))) OR ((glucophage) AND (glucophage[MeSH Terms])) OR ((biomet) AND (biomet[MeSH Terms]))) OR ((body weight loss) AND (body weight loss[MeSH Terms]))) OR ((ethanol) AND (ethanol[MeSH Terms]))) OR ((folate) AND (folate[MeSH Terms]))) OR ((walking) AND (walking[MeSH Terms]))) OR ((meditation) AND (meditation[MeSH Terms]))) OR ((hypnosis) AND (hypnosis[MeSH Terms]))) OR ((jogging) AND (jogging[MeSH Terms]))) OR ((yoga) AND (yoga[MeSH Terms]))) OR (((((frequency) AND (frequency[MeSH Terms])) OR ((compliance) AND (compliance[MeSH Terms]))) OR (timing)) AND ((intercourse) AND (intercourse[MeSH Terms])))) OR ((physical activit*)) OR ((exercis*) AND (exercis*[MeSH Terms]))) OR (BMI)) OR ((Body mass index) AND (Body mass index[MeSH Terms]))) OR ((overweight) AND (overweight[MeSH Terms]))) OR ((obes*) AND (obes*[MeSH Terms]))) OR ((weight) AND (weight[MeSH Terms]))) OR ((iodine) AND (iodine[MeSH Terms]))) OR ((folic acid) AND (folic acid[MeSH Terms]))) OR ((vitamin) AND (vitamin[MeSH Terms]))) OR ((nutraceutical) AND (nutraceutical[MeSH Terms]))) OR ((low fat diet) AND (low fat diet[MeSH Terms]))) OR ((diet therapy) AND (diet therapy[MeSH Terms]))) OR ((healthy diet) AND (healthy diet[MeSH Terms]))) OR (diet supplementation)) OR ((low carbohydrate diet) AND (low carbohydrate diet[MeSH Terms]))) OR (diet restriction)) OR ((low calory diet) AND (low calory diet[MeSH Terms]))) OR ((coffee) AND (coffee[MeSH Terms]))) OR ((caffeine) AND (caffeine[MeSH Terms]))) OR ((alcohol) AND (alcohol[MeSH Terms]))) OR ((tobacco cessation) AND (tobacco

cessation[MeSH Terms]))) OR ((marijuana) AND (marijuana[MeSH Terms]))) OR ((cocaine) AND (cocaine[MeSH Terms]))) OR ((recreational drug) AND (recreational drug[MeSH Terms]))) OR ((drug abuse) AND (drug abuse[MeSH Terms]))) OR ((street drug) AND (street drug[MeSH Terms]))) OR (tobacco reduction)) OR ((tobacco use) AND (tobacco use[MeSH Terms]))) OR ((reproductive behavior) AND (reproductive behavior[MeSH Terms]))) OR ((cannabis) AND (cannabis[MeSH Terms]))) OR ((smoking) AND (smoking[MeSH Terms]))) OR ((health behavior) AND (health behavior[MeSH Terms]))) OR ((drinking behavior) AND (drinking behavior[MeSH Terms]))) OR ((patient education) AND (patient education[MeSH Terms]))) OR (decision support)) OR ((behavior therapy) AND (behavior therapy[MeSH Terms]))) OR ((attitude to health) AND (attitude to health[MeSH Terms]))) OR ((life style) AND (life style[MeSH Terms]))) OR ((lifestyle) AND (lifestyle[MeSH Terms]))) OR ((e-counseling) AND (e-counseling[MeSH Terms]))) OR ((counseling) AND (counseling[MeSH Terms]))) OR ((Intra-uterine insemination) AND (Intra-uterine insemination[MeSH Terms]))) OR (IUI)) OR ((Intrauterine insemination) AND (Intrauterine insemination[MeSH Terms]))) OR ((Assisted reproduction) AND (Assisted reproduction[MeSH Terms]))) OR ((assisted reproductive technology) AND (assisted reproductive technology[MeSH Terms]))) OR (ART)) OR (ICSI)) OR ((Sperm Injection) AND (Sperm Injection[MeSH Terms]))) OR ((in-vitro fertilisation) AND (in-vitro fertilisation[MeSH Terms]))) OR ((in vitro fertilisation) AND (in vitro fertilisation[MeSH Terms]))) OR ((invitro fertilisation) AND (invitro fertilisation[MeSH Terms]))) OR ((in-vitro fertilization) AND (in-vitro fertilization[MeSH Terms]))) OR ((in vitro fertilization) AND (in vitro fertilization[MeSH Terms]))) OR ((invitro fertilization) AND (invitro fertilization[MeSH Terms]))) OR (ivf)) OR ((Fertilization in Vitro) AND (Fertilization in Vitro[MeSH Terms]))) OR ((follitropin) AND (follitropin[MeSH Terms]))) OR ((Follicle-stimulating hormone) AND (Follicle-stimulating hormone[MeSH Terms]))) OR ((lutropin) AND (lutropin[MeSH Terms]))) OR ((luteinising hormone) AND (luteinising hormone[MeSH Terms]))) OR ((Luteinizing hormone) AND (Luteinizing hormone[MeSH Terms]))) OR (((gn) OR (LH)) OR (FSH))) OR ((superovulation) AND (superovulation[MeSH Terms]))) OR ((Gonadotrophin) AND (Gonadotrophin[MeSH Terms]))) OR ((Gonadotropin) AND (Gonadotropin[MeSH Terms]))) OR (Follutein)) OR (Profasi)) OR ((Pregnyl) AND (Pregnyl[MeSH Terms]))) OR (Ovidrel)) OR (Novarel)) OR ((Human chorionic gonadotrophin) AND (Human chorionic gonadotrophin[MeSH Terms]))) OR (hCG)) OR ((Human chorionic gonadotropin) AND (Human chorionic gonadotropin[MeSH Terms]))) OR ((Bromocryptin) AND (Bromocryptin[MeSH Terms]))) OR (cb 154)) OR ((CB-154) AND (CB-154[MeSH Terms]))) OR (cycloset)) OR ((Parlodel) AND (Parlodel[MeSH Terms]))) OR ((dopamine agonist) AND (dopamine agonist[MeSH Terms]))) OR ((Bromocriptine) AND (Bromocriptine[MeSH Terms]))) OR (riomet)) OR ((biomet) AND (biomet[MeSH Terms]))))) AND (((infertili*) AND (infertili*[MeSH Terms])) OR (subfertili*)

Scopus (n = 1362)

1. Search within Articles title, asbtract, keywords: (((((((((economic evaluation) AND (economic evaluation)) OR ((Cost benefit analysis) AND (Cost benefit analysis))) OR ((Cost utility analysis) AND (Cost utility analysis))) OR (cua)) OR (cba)) OR (cma)) OR ((Cost minimization analysis) AND (Cost minimization analysis))) OR ((Cost-effective analysis) OR (cea))) AND ((((((((((((((((((gonadotropin-releasing hormone) AND

(gonadotropin-releasing hormone)) OR ((induction of ovulation) AND (induction of ovulation))) OR (fertility drug)) OR ((fertility agent) AND (fertility agent))) OR (ovarian hyperstimulation)) OR ((ovarian stimulation) AND (ovarian stimulation))) OR (Fortamet)) OR (mfm)) OR ((metformin) AND (metformin))) OR ((Femara) AND (Femara))) OR (AI)) OR ((aromatase inhibitor) AND (aromatase inhibitor))) OR ((non-steroidal inhibitor of aromatase) AND (non-steroidal inhibitor of aromatase))) OR ((CGS 20267) OR (CGS 20267))) OR ((letrozole) AND (letrozole))) OR ((selective estrogen receptor modulator) AND (selective estrogen receptor modulator))) OR ((clomid) OR (clomid))) OR ((cc) OR (serm))) OR ((Clomifene citrate) AND (Clomifene citrate))) OR (((glucophage) AND (glucophage)) OR ((biomet) AND (biomet))) OR ((body weight loss) AND (body weight loss))) OR ((ethanol) AND (ethanol))) OR ((folate) AND (folate))) OR ((walking) AND (walking))) OR ((meditation) AND (meditation))) OR ((hypnosis) AND (hypnosis))) OR ((jogging) AND (jogging))) OR ((yoga) AND (yoga))) OR (((((frequency) AND (frequency)) OR ((compliance) AND (compliance))) OR (timing)) AND ((intercourse) AND (intercourse)))) OR ((physical activit*) AND (physical activit*))) OR ((exercis*) AND (exercis*))) OR (BMI)) OR ((Body mass index) AND (Body mass index))) OR ((overweight) AND (overweight))) OR ((obes*) AND (obes*))) OR ((weight) AND (weight))) OR ((iodine) AND (iodine))) OR ((folic acid) AND (folic acid))) OR ((vitamin) AND (vitamin))) OR ((nutraceutical) AND (nutraceutical))) OR ((low fat diet) AND (low fat diet))) OR ((diet therapy) AND (diet therapy))) OR ((healthy diet) AND (healthy diet))) OR (diet supplementation)) OR ((low carbohydrate diet) AND (low carbohydrate diet))) OR (diet restriction)) OR ((low calory diet) AND (low calory diet))) OR ((coffee) AND (coffee))) OR ((caffeine) AND (caffeine))) OR ((alcohol) AND (alcohol))) OR ((tobacco cessation) AND (tobacco cessation))) OR ((marijuana) AND (marijuana))) OR ((cocaine) AND (cocaine))) OR ((recreational drug) AND (recreational drug))) OR ((drug abuse) AND (drug abuse))) OR ((street drug) AND (street drug))) OR (tobacco reduction)) OR ((tobacco use) AND (tobacco use))) OR ((reproductive behavior) AND (reproductive behavior))) OR ((cannabis) AND (cannabis))) OR ((smoking) AND (smoking))) OR ((health behavior) AND (health behavior))) OR ((drinking behavior) AND (drinking behavior))) OR ((patient education) AND (patient education))) OR (decision support)) OR ((behavior therapy) AND (behavior therapy))) OR ((attitude to health) AND (attitude to health))) OR ((life style) AND (life style))) OR ((lifestyle) AND (lifestyle))) OR ((e-counseling) AND (e-counseling))) OR ((counseling) AND (counseling))) OR ((Intra-uterine insemination) AND (Intra-uterine insemination))) OR (IUI)) OR ((Intrauterine insemination) AND (Intrauterine insemination))) OR ((Assisted reproduction) AND (Assisted reproduction))) OR ((assisted reproductive technology) AND (assisted reproductive technology))) OR (ART)) OR (ICSI)) OR ((Sperm Injection) AND (Sperm Injection))) OR ((in-vitro fertilisation) AND (in-vitro fertilisation))) OR ((in vitro fertilisation) AND (in vitro fertilisation))) OR ((invitro fertilisation) AND (invitro fertilisation))) OR ((in-vitro fertilization) AND (in-vitro fertilization))) OR ((in vitro fertilization) AND (in vitro fertilization))) OR ((invitro fertilization) AND (invitro fertilization))) OR (ivf)) OR ((Fertilization in Vitro) AND (Fertilization in Vitro))) OR ((follitropin) AND (follitropin))) OR ((Follicle-stimulating hormone) AND (Follicle-stimulating hormone))) OR ((lutropin) AND (lutropin))) OR ((luteinising hormone) AND (luteinising hormone))) OR ((Luteinizing hormone) AND (Luteinizing hormone))) OR (((gn) OR (LH)) OR (FSH))) OR ((superovulation) AND

(superovulation))) OR ((Gonadotrophin) AND (Gonadotrophin))) OR ((Gonadotropin) AND (Gonadotropin))) OR (Follutein)) OR (Profasi)) OR ((Pregnyl) AND (Pregnyl))) OR (Ovidrel)) OR (Novarel)) OR ((Human chorionic gonadotrophin) AND (Human chorionic gonadotrophin))) OR (hCG)) OR ((Human chorionic gonadotropin) AND (Human chorionic gonadotropin))) OR ((Bromocryptin) AND (Bromocryptin))) OR (cb 154)) OR ((CB-154) AND (CB-154))) OR (cycloset)) OR ((Parlodel) AND (Parlodel))) OR ((dopamine agonist) AND (dopamine agonist))) OR ((Bromocriptine) AND (Bromocriptine))) OR (riomet)) OR ((biomet) AND (biomet))) OR ((glucophage) AND (glucophage))))) AND (((infertili*) AND (infertili*)) OR (subfertili*))
