# Supplementary material for: Economic evaluation of lifestyle interventions in infertility management: A systematic review
Source: PLoS One. 2024 Aug 23;19(8):e0306419. doi: 10.1371/journal.pone.0306419 (PMC11343367; doi:10.1371/journal.pone.0306419)
Supplement: S3 File — (PDF) [file pone.0306419.s003.pdf]

Supplementary data 3: quality assessment of included studies

Author, Year	Was a well-defined question posed?	Was a comprehensive description of the competing alternatives offered?	Was the evidence of the effectiveness of the program offered?	Were all important and relevant costs and consequences identified?	Were all important and relevant costs and consequences measured accurately?	Were all important and relevant costs and consequences have been properly valued?	Were the costs and consequences adjusted for different times?	Was an incremental analysis of costs and consequences of competing alternatives done?	Was the effect of uncertainty (sensitivity analysis) investigated in estimating the costs and consequences?	Were the presentation and analysis of all issues related to users of the results included?	Conclusions
Mori, 2021[12]	yes	yes	yes	yes	yes	yes	no	no	no	yes	7
Oostingh, 2019 [9]	yes	yes	yes	yes	yes	yes	no	yes	yes	yes	9
Sim, 2013 [11,17]	yes	yes	yes	yes	yes	yes	no	no	no	yes	7
Steegers-Theunissen, 2020 [8]	yes	yes	yes	yes	yes	yes	no	no	no	yes	7
van Oers, 2017 [16]	yes	yes	yes	yes	yes	yes	no	no	no	yes	7
